# Responses of Soil Fungal Populations and Communities to the Thinning of *Cryptomeria Japonica* Forests

**DOI:** 10.1264/jsme2.ME15127

**Published:** 2016-02-20

**Authors:** Wan-Rou Lin, Pi-Han Wang, Wen-Cheng Chen, Chao-Ming Lai, Richard Scott Winder

**Affiliations:** 1 Department of Life Sciences, Tunghai UniversityNo. 1727, Sec. 4, Taiwan Boulevard, Taichung 40704Taiwan; 2 Department of Agricultural Chemistry, National Taiwan UniversityNo. 1, Sec. 4, Roosevelt Road, Taipei, Taiwan 10617; 3 Natural Resources Canada, Canadian Forest Service, Pacific Forestry Centre506 West Burnside Road, Victoria, British Columbia V8Z 1M5Canada

**Keywords:** plate count, forest soil, tree thinning, microbial ecology, fungal community

## Abstract

Forest management activities, such as tree thinning, alter forest ecology, including key components of forest ecosystems, including fungal communities. In the present study, we investigate the effects of forest thinning intensity on the populations and structures of fungal soil communities in the *Cryptomeria japonica* forests of central Taiwan as well as the dynamics of soil fungi communities in these forests after a thinning disturbance. Although the populations of soil fungi significantly increased in the first 6 months after thinning, these increases had subsided by 9 months. This pulse was attributed to a transient increase in the populations of rapid colonizers. A multiple regression analysis positively correlated fungal populations with organic matter content and cellulase activity. Thinning initially provided large amounts of fresh leaves and roots as nutrient-rich substrates for soil fungi. Denaturing gradient gel electrophoresis (DGGE) profiles indicated that soil fungal communities significantly differed among plots with 0% (control), 25%, and 50% tree thinning in the first 21 months post-thinning, with no significant differences being observed after 21 months. The fungal communities of these forest soils also changed with the seasons, and an interactive relationship was detected between seasons and treatments. Seasonal variations in fungal communities were the most pronounced after 50% tree thinning. The results of the present study demonstrate that the soil fungi of Taiwanese *C. japonica* forests are very sensitive to thinning disturbances, but recover stability after a relatively short period of time.

Forest management activities, such as tree harvesting, forest thinning, and clear cutting, are key disturbances in many forest ecosystems. Thinning, a common way to manage forests, increases tree stem diameter (60), crown size (59), and woody litter fall (19). It has also been shown to enhance the growth rate of the remaining trees (29) and decrease their mortality rate (9). However, thinning also influences the understory organisms in forest ecosystems (6, 42, 63), thereby affecting the functions of the ecosystem (7).

Bacteria, fungi, and other soil microorganisms play important roles in the ecological processes and nutrient dynamics of forest ecosystems (21, 44). Fungi account for the majority of the microbial biomass in forest soil and convert recalcitrant organic material into forms useable by other organisms (22). Most studies that have examined the influence of tree thinning on these organisms have focused on the impact of this major disturbance on soil bacteria or ectomycorrhizal fungi (57), and concluded that the effects of thinning on soil microorganisms appear to be complex. Thinning has been shown to significantly change soil microbial communities in a pine (*Pinus* spp.) forest (49), Jarrah (*Eucalyptus marginata*) forest (18), and *Chamaecyparis formosensis* forest (45). Previous studies reported that forest thinning did not significantly affect carbon associated with the microbial biomass, enzyme activity, soil respiration (49), or soil fungal communities (35). Grayston and Rennenberg (31) demonstrated that the influence of heavy thinning on the microbial biomass varied spatially. Levy-Booth and Winder (44) also found that the impact of thinning on free-living diazotrophic and denitrifying bacteria varied spatially, making environmental trends for these microbial communities difficult to discern.

In addition to the disturbances described above, temporal and seasonal effects have also been shown to influence soil microorganisms. For example, the phospholipid fatty acid (PLFA) profiles of microbial communities were found to be altered by changes in seasons (5, 8, 10, 33). Moreover, fungal communities associated with oak rhizospheres and grassland soils are known to be seasonally dynamic with temporal turnover (39, 65).

Most studies on the impact of thinning on soil microbes have compared microbial communities in forests 3 to 45 years post-thinning (3, 4, 11, 18, 34, 35, 49). Long-term assessments have generally not focused on short-term and seasonal dynamics. A previous study on the short-term responses of soil decomposer communities in a boreal spruce (*Picea abies*) forest found that the microbial biomass was reduced by clear cutting, with a corresponding structural change in the community as measured by PLFA patterns. However, selective felling had no discernable impact (58).

A clearer understanding of the short-term effects of thinning on microbial communities in forest soils will address an important knowledge gap. Fungi play important roles in recycling important chemical elements in terrestrial ecosystems, making nutrients available during critical plant growth phases and assisting in primary production. In addition to their role in nutrient cycling, fungi also participate in ecological food webs (22) and are key determinants in the biodiversity of understory plant communities (54, 64). Their activity may affect the success of seedling establishment and growth during the critical period following tree removal (36). Since soil fungi play a crucial role in the forest community structure and productivity, it is important to elucidate the responses of these organisms to disturbances as well as their dynamics in the period that follows.

We herein examined the responses of soil fungi to thinning in a Japanese cedar (*Cryptomeria japonica*) forest plantation in central Taiwan. The objectives of the present study were to 1) investigate thinning effects on soil microfungal populations and communities in *C. japonica* plantations; 2) evaluate changes corresponding to thinning intensity; and 3) determine the duration of any thinning effects influencing fungal communities in the soils of these forests. Plate counts and denaturing gradient gel electrophoresis (DGGE) were used as relatively simple methods to monitor fluctuations in soil fungal populations and describe changes in their genetic diversities and communities after thinning. These methods were chosen because of their relative cost efficiency and ability to detect major community shifts (68) without saturating the acquired datasets.

## Materials and Methods

### Study site

The study site is located in the Luan-Da forest management district, Nantou County, in central Taiwan, in a 40-year old plantation of *C. japonica*. The site ranges from 23°28′ to 23°55′N latitude and from 120°48′ to 121°09′E longitude. The elevation ranges from 1,275 to 1,500 m. According to records from the nearby Sun Moon Lake Weather Station (23°53′N, 120°54′E), average annual air temperature and rainfall are 19.2°C and 2,401.9 mm, respectively (Climate data from Sun Moon Lake Meteorological Observatory, http://www.cwb.gov.tw/V7/climate/monthlyMean/Taiwan_tx.htm, accessed 20 January 2011). Most rainfall at the site occurs during the summer, from June to September. Some rainfall also occurs during the spring, from March to May; there is no obvious dry season.

**Fig. 1:**
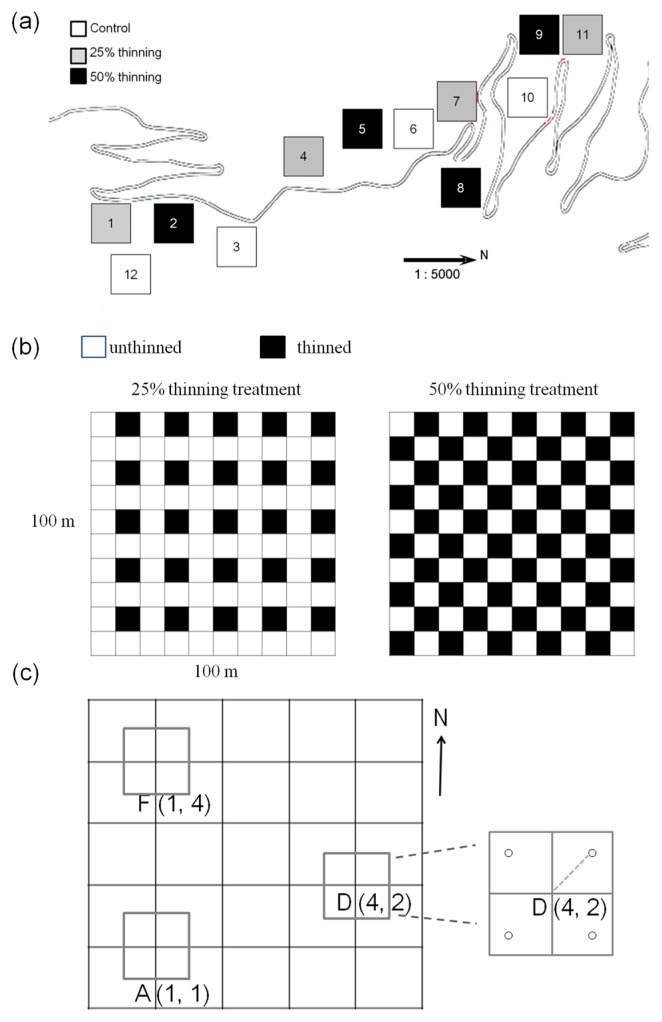
(a) Twelve 1-ha plots (100 × 100 m) with 3 treatments (control and two thinning intensities) of 4 replicates distributed in a plantation of Japanese cedar, *Cryptomeria japonica* in central Taiwan. (b) Schematic of the thinning method comparing the arrangement of thinned (completely harvested) treatment areas corresponding to 25% thinning and 50% thinning treatments. The 100×100 m plots were divided into sections using a grid with 10-m intervals. In the thinned plots, all trees within a 10×10-m section (black quadrats in the schematic) were thinned. (c) A schematic showing the arrangement of three sampling sites (A, D, and F) within each 1-ha plot. There were four sampling points in each sampling site in the center of each of the four quadrants of the sampling site. A soil sample was taken from the organic soil layer at each of the four sample points. These four subsamples were mixed. Each plot contained three replicated soil samples. Soil samples were taken from all of the plots periodically starting in October 2008.

Twelve 1-ha permanent plots (100×100 m) were established (Fig. 1A) for the long-term monitoring of biodiversity dynamics. The twelve plots were divided into four blocks, which had slightly different elevations on a slope. Each block had three plots, and each plot was randomly assigned a control (0%), 25%, or 50% thinning treatment (Fig. 1A). Each plot was divided into one hundred 10×10 m grids (Fig. 1B). The x and y coordinates of the plot were staked at 20-m and 10-m intervals. Thinning treatments consisted of tree removal in alternating quadrats, as indicated in Fig. 1B. In treatment plots with 25% thinning, trees in one 10-m quadrant within each 20-m quadrant were cut, while in the 50% thinning plots, trees in two 10-m quadrants within each 20-m quadrant were thinned (Fig. 1B). Thinning was performed using chainsaws in August 2007 and finished in October 2007. Most slash and coarse woody debris was removed, and some minor fragmentary debris remained.

### Soil sampling

Soil samples were collected in two ways. In the cultural analysis of fungal populations, soil samples were collected seasonally between January 2008 and August 2009. In each plot, 100 g topsoil (depth of 15 cm, diameter of *ca.* 15 cm) samples from four 10×10 m random quadrants were collected using a trowel and pooled together. There was one mixed soil sample per plot, resulting in each treatment providing four replicate soil samples. Soil samples were sieved with a 2-mm mesh and stored at 4°C until analyzed.

In the molecular analysis of the fungal communities in the soil, we selected three sampling sites (Fig. 1C) in each plot. Each sampling site had four sample points (Fig. 1C). Soil samples were collected from the topsoil of each sampling point and were mixed into one sample; this provided three replicate pooled soil samples from each plot. During the first year, subsequent to thinning (October 2008 to August 2009), soil samples were taken seasonally. In order to determine the long-term impact on soil microbe communities, samples were also taken annually in the second (October 2009) and third (October 2010) years after thinning. Prior to DNA extraction, each soil sample was sieved and stored as described above.

### Abundance of fungal populations

Sieved soil (10 g) was added to 90 mL of 0.1% water agar, which was then mixed well. Soil suspensions were serially diluted with 0.1% water agar. Diluted suspensions were spread on Rose Bengal agar (13). Each assessment was performed in triplicate. The plates were incubated at 25°C, and fungal colonies were counted after 5 d. Some colonies were examined with a microscope to visually characterize the most abundant types.

### DNA extraction and amplification

DNA was extracted from 0.25 g of each soil sample using a PowerSoil^®^ DNA Combo Kit (Mo Bio Laboratories, Inc., CA, USA). Fungal rDNA internally transcribed spacer (ITS) regions were amplified twice using a semi-nested PCR amplification method. In the first PCR amplification, fungal-specific ITS1F (26) and ITS4 (67) primers were used to amplify the ITS regions. PCR reactions were performed using a PCR thermocycler (Biometra, Gottingen, UK). The 25-μL PCR mixture included 10 ng of template DNA, 250 μM of each primer, 250 μM of dNTP, 1 U Taq DNA polymerase (Fermentas, USA), 2.5 mM MgCl_2_ (Fermentas, USA), and 2.5 μL of 10× buffer (Fermentas, USA). The amplification protocol consisted of one denaturation step at 94°C for 5 min, 39 cycles of denaturation at 94°C for 1 min, annealing at 50°C for 1 min, extension at 72°C for 1 min, and a final extension at 72°C for 5 min. First-round PCR products were used as template DNA in the second PCR amplification, which used an ITS2 primer (67) and ITS1F fungal-specific primer (26) with a 40-base GC-clamp attached to the 5′ end of the primer to aid in DGGE. The PCR mixtures and program were identical to the first PCR protocol. Agarose gel electrophoresis was used to evaluate and verify the quality and amount of PCR products.

### DGGE analysis

In order to analyze the genetic diversities and structures of the soil fungal communities, all PCR products were analyzed with DGGE using the DCODE^™^ Universal Mutation Detection System (Bio-Rad Laboratories Limited). DGGE was performed using 8% polyacrylamide gels (40% acrylamide/bis-acrylamide [37:5:1] Bio-Rad stock solution) prepared with a 20% (1.4 M urea, 8% [v/v] formamide) to 45% (3.85 M urea, 22% [v/v] formamide) vertical denaturing gradient according to the manufacturer’s suggested protocol. Electrophoresis was performed in 1X tris-acetate buffer at 60°C, using 70 volts for 16 h. Subsequent to electrophoresis, the gel was stained with SYBR gold (Molecular Probes, USA) in darkness at room temperature (*ca.* 20°C) for 30 min, rinsed once in deionized water, and photographed under UV light.

### Data analysis

Measurements of the abundance of fungi in soils were subjected to a variance analysis (ANOVA) in order to determine the effects of forest thinning. Tukey’s test determined differences among treatments. Microbial data were correlated with the physical soil properties and enzyme activities published by Chung *et al.* (14). Physical soil properties and enzyme activities were sampled at points corresponding to microbial soil samples. Soil measurements included the pH value, organic matter, total nitrogen, inorganic nitrogen, and available phosphorus. Soil enzyme data included the activities of cellulase, glucosaminidase, acid phosphatase, arylsulfatase, and dehydrogenase. Briefly, pH ranged from 3.89 to 4.14; the organic matter content ranged from 21.2 to 34.1 (g kg^−1^ dry soil); total nitrogen ranged from 0.68 to 1.72 (g kg^−1^ dry soil), inorganic nitrogen ranged from 248 to 539 (mg kg^−1^ dry soil); the available phosphorus content ranged from 2.41 to 3.02 (mg kg^−1^ dry soil); cellulase activity ranged from 0.11 to 0.32 (μmol g^−1^); glucosaminidase activity ranged from 1.20 to 1.81 (μmol g^−1^); acid phosphatase ranged from 21.94 to 30.38 (μmol g^−1^); arylsulfatase ranged from 7.85 to 12.66 (μmol g^−1^); and dehydrogenase ranged from 0.06 to 0.12 (μmol g^−1^). The relationships between the abundance of soil fungi and physiochemical soil properties were analyzed using a multiple linear regression model.

Image processing software (Quantity One, Bio-Rad, USA) was used to analyze and quantify bands on DGGE gels. The band intensity in each sample was recorded as a binary matrix. The binary matrix was used to calculate the Bray-Curtis Similarity Index (16) and construct nonparametric multi-dimensional scaling (MDS) plots using Primer 6 software (version 6. 1. 15; Primer-E Ltd., United Kingdom). The PERMANOVA plug-in (version 1.0.5) of the Primer package was used to statistically test any observed differences. A repeated measurement design permutational multivariate analysis of variance (PERMANOVA) was calculated using time (including seasons and years) and the thinning treatment as fixed factors and block as a random factor in order to compare soil fungal community compositions over time. If significant differences were detected for time, treatments, or interactions, pairwise PERMANOVA was performed on the respective term for each level of the factor. In all of the PERMANOVAs, a maximum of 9999 random permutations was performed.

## Results

### Populations of soil fungi

**Fig. 2:**
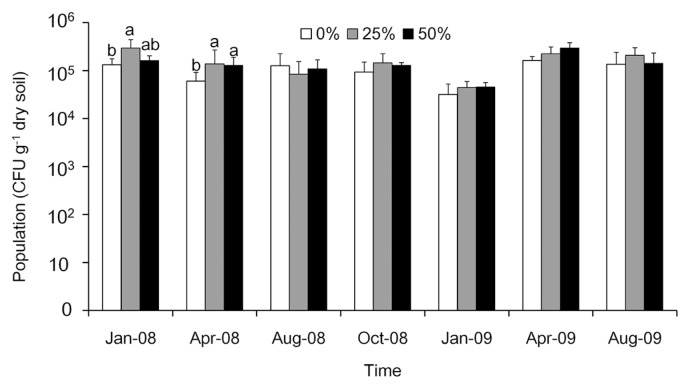
Abundance of fungal populations in soil samples collected after control, 25%, and 50% thinning treatments in a *Cryptomeria japonica* plantation in central Taiwan between January 2008 and August 2009. Bars with the same letter are not significantly different according to Tukey’s multi-range test (*P* > 0.05). In January 2008 and April 2008, thinned plots had significantly (*P* = 0.045) higher fungal counts than those of the controls.

The study sites were thinned in October 2007. Fungal populations in January 2008 and April 2008 were 1.1×10^5^ to 2.1×10^5^ and 4.7×10^4^ to 1.1×10^5^ colony forming units (CFU) g^−1^ of dry soil, respectively (Fig. 2); thinned plots had significantly (*P* = 0.045) higher fungal counts than those of the controls. In August 2008, October 2008, January 2009, April 2009, and August 2009, the abundances of fungal populations ranged from 6.0×10^4^ to 8.5×10^4^, 9.3×10^4^ to 1.4×10^5^, 1.5×10^5^ to 3.3×10^5^, 1.6×10^5^ to 3.0×10^5^, and 1.3×10^5^ to 2.0×10^5^ CFU g^−1^ dry soil, respectively (Fig. 2). No significant differences were observed in abundance among the treatments between August 2008 and August 2009 (*P* < 0.05). Thinning increased the fungal population in the first six months after thinning. However, after 10 months, no significant differences were noted in abundance among the treatments (*P* = 0.7). Dominant fungi included *Penicillium* spp., *Fusarium* spp., *Trichoderma* spp., and Zygomycetes.

The relationships between soil fungal populations and soil properties were analyzed by a multiple regression analysis. The amount of organic matter and the activity of cellulase were identified as good estimators of the soil fungal population (*R*^2^ = 0.801; Adjusted *R*^2^ = 0.713). A regression analysis indicated that the population of soil fungi positively correlated with the amount of organic matter (*β* =1.92×10^5^, *P* < 0.001) and activity of cellulase (*β* =1.20×10^5^, *P* < 0.05). Other factors, including the water content, pH value, total nitrogen, inorganic nitrogen, the available phosphorus content, and soil temperature were not associated with the soil fungal population.

### Community compositions of soil fungi

**Fig. 3:**
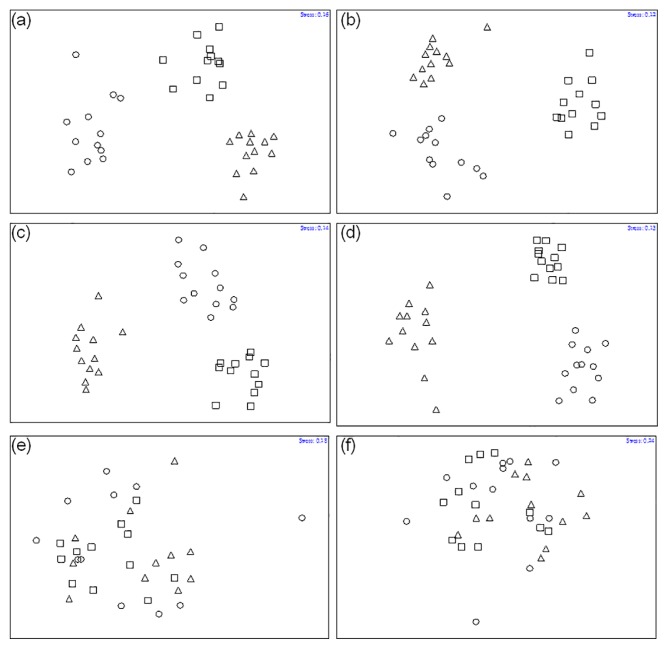
Multidimensional scaling of DGGE profiles of soil samples collected from three thinning treatments in a *Cryptomeria japonica* plantation in central Taiwan. Symbols indicate different levels of thinning treatments: circles (○) = 0% thinning (controls); triangles (△) = 25% thinning; and squares (□) = 50% thinning. (a) Samples collected in October 2008 (12 months post-thinning). (b) Samples collected in January 2009 (15 months post-thinning). (c) Samples collected in April 2009 (18 months post-thinning). (d) Samples collected August 2009 (21 months post-thinning). (e) Samples collected October 2009 (24 months post-thinning). (f) Samples collected October 2010 (36 months post-thinning).

In MDS plotting, the compositions of fungal communities in the plantation soils were grouped separately according to the corresponding thinning treatments at 12–21 months post-thinning (Fig. 3, panels A–D); there was no significant (*P* = 0.15) clustering of these communities at 24 months post-thinning (Fig. 3, Panels E–F). PERMANOVA showed that the compositions of soil fungal communities were significantly different (*P* = 0.0007) due to thinning treatments (Table 1). Pairwise comparisons of DGGE gels revealed that thinning levels significantly (*P* < 0.05) influenced the composition of fungal communities in all treatments at 21 months post-thinning (data not shown); however, there was no significant effect (*P* > 0.1) of thinning on these communities in any of the thinning treatments by 24 months post-thinning (data not shown).

**Fig. 4:**
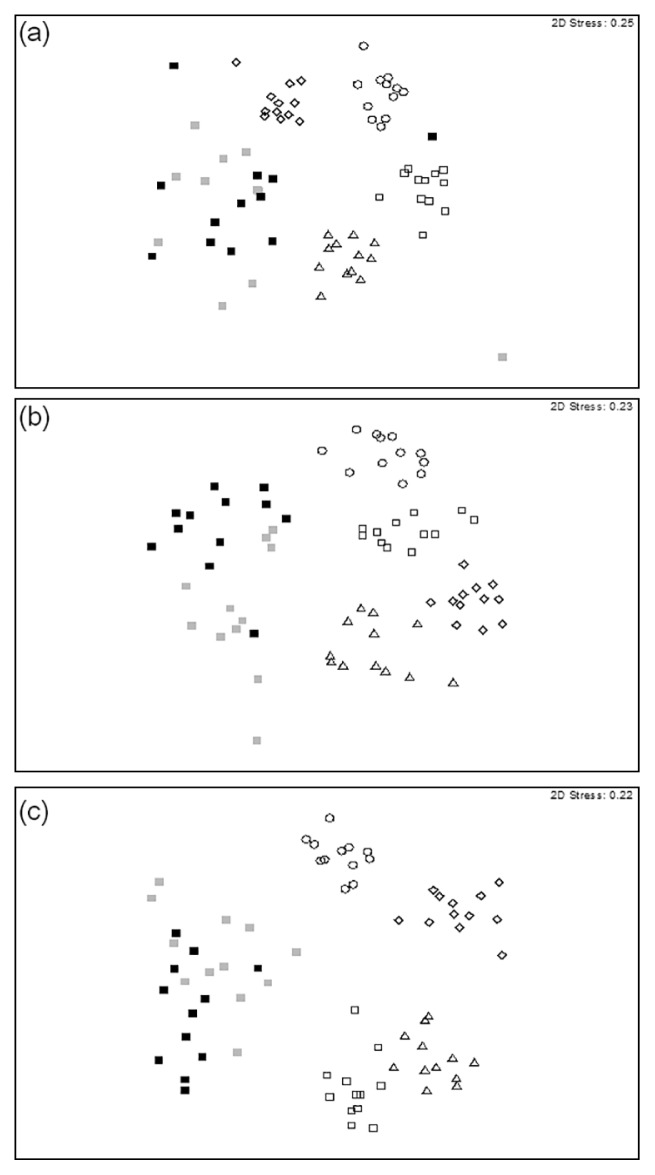
Multidimensional scaling of seasonal DGGE profiles of soil samples collected from variously thinned plots in a *Cryptomeria japonica* plantation in central Taiwan. Symbols correspond to samples from four seasons: circle (○) = spring; triangles (△) = summer; squares (□) = fall; and diamonds (⋄) = winter. Colors correspond to samples from different sampling years: white =2008; light gray = 2009; black = 2010. (a) Samples from plots with 0% thinning (controls). (b) Samples from plots with 25% thinning. (c) Samples from plots with 50% thinning.

**Fig. 5:**
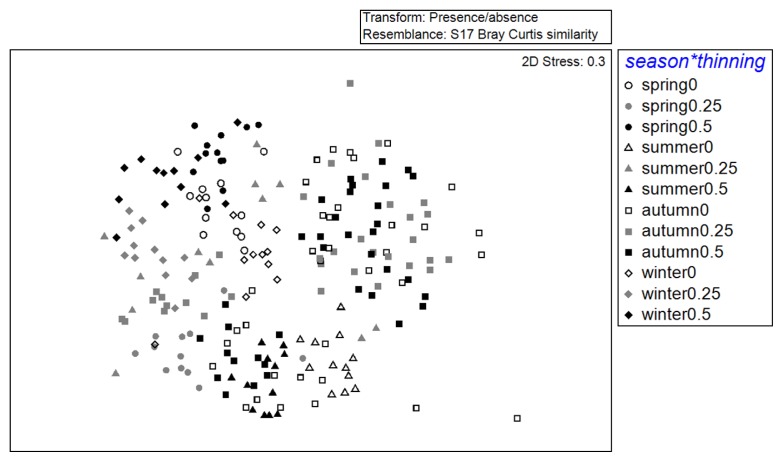
Multidimensional scaling of seasonal DGGE profiles of soil samples collected from variously thinned plots in a *Cryptomeria japonica* plantation in central Taiwan in 2008. Symbols correspond to the sample season: circles (○) = spring; triangles (△) = summer; squares (□) = fall; and diamonds (⋄) = winter. Colors correspond to samples from different thinning treatments: white = samples from control plots; light gray = samples from 25% thinned plots; black = samples from 50% thinned plots.

DGGE patterns were also analyzed in order to examine the temporal (seasonal) dynamics of soil fungal communities among different thinning treatments using the MDS and PERMANOVA tests. Fig. 4 shows distinct seasonal variations in fungal community compositions with the control (Fig. 4A), 25% thinning (Fig. 4B), and 50% thinning treatments (Fig. 4C). The results of the PERMANOVA tests showed that the compositions of fungal communities in these soils varied significantly (*P* = 0.0001) among seasons (Table 1), and pairwise comparisons showed that the seasonal effect was consistently observed throughout the study period because significant (*P* < 0.05) differences were detected in all pairwise tests among seasons. A season × treatment interaction was found for all treatments (Table 1). The 50% thinning treatment increased the variability of fungal communities between seasons. There was a higher DGGE pattern Bray-Curtis dissimilarity between seasons in the 50% thinning treatment than in the comparison among seasons in the control plots (Fig. 5, Table 2).

The dissimilarity in DGGE patterns among treatment pair comparisons in 2008 was higher than that in 2009 or 2010 (Table 3), indicating that differences between treatment and control plots decreased by the second year post-thinning and also that the influence of thinning on fungal communities had diminished.

## Discussion

We herein evaluated the effects of thinning disturbances and seasons on the abundance of soil fungi and the structures of these communities in forest soils using cultural methods and DGGE profiles. Thinning was found to increase soil fungal populations within 6 months of the disturbance, and also changed soil fungal community structures in the short term. Other studies have reported similar impacts, with thinning heightening soil respiration (51) and microbial biomass/activity (32), and changing the structures of soil microbe communities (49). Green leaves contain nutrients that are more easily decomposed than fallen leaves (27), providing extra available carbon, nitrogen, phosphorus, and other nutrients for microbial growth (47). Previous studies reported that early colonizer taxa readily respond to decomposable substrates from logging slash, leaf litter, fallen branches (52, 56), and dying roots. In the present study, thinning provided green fallen leaves, shoots, and dying and dead roots as nutritive substrates for soil saprotrophic microorganisms. The fungal population positively correlated with the organic matter content, and this was reflected in the correlation with cellulase activity.

In forest ecosystems, fungi convert recalcitrant organic material into forms useable by other organisms. In balsam fir stands, for example, thinning was reported to significantly increase organic matter decomposition, as indicated by mass loss in cellulose bags from 6 to 18 months post-thinning; larger fungal populations were identified as the cause of increased cellulose decomposition (62). In the *C. japonica* plantation, thinning increased the fungal population in the first six months post-thinning, and soil fungal abundance positively correlated with the amount of organic matter and the activity of cellulase.

We presume that the return of fungal populations to their initial levels of abundance was caused by the depletion of the easily decomposable substrates provided by logging slash, dead stumps, and roots. As substrate availability diminished, more oligotrophic taxa may have metabolized the remaining recalcitrant organic carbon pools and likely replaced the early colonizers taxa (25, 52, 56). This shift in the soil fungal community may also have been compounded by other contributory factors, such as changes in temperature and humidity and exchangeable cations in the soil within the local microenvironment. In other studies that were performed at the same study site, air and soil temperatures increased and relative humidity decreased during the first year post-thinning; these thinning effects gradually diminished during the second year (12). Other researchers also found that the total number of exchangeable cations increased in site soils during the first year post-thinning, and levels subsequently reverted to their original level after 16 months (46). Thinning also creates forest gaps and increases the available light to the forest. Tree cover has been reported to have an impact on soil microbial community structures in forests because it influences the microclimate and physicochemical properties of soil (11, 17, 24, 38, 49, 66, 70).

The structure of the fungal communities in plantation soils was also shown to vary seasonally. Moreover, we found that thinning treatments emphasized the seasonal dynamics of the soil fungal community. Seasonal differences in these communities may be caused by seasonal variations in climate, soil properties, substrate availability, plant productivity, and litter deposition throughout the year (30). As environmental changes increase, corresponding increases occur in fluctuations in fungal community structures in forest soils.

Forest ecosystems are complex. While fungal communities, including pathogens and mycorrhizal species, may be the major drivers of plant community structures and forest biodiversity (69), plant species diversity is also known to conversely influence fungal community structures (20). Therefore, the changes observed in fungal communities in the present study may also reflect those in the composition of understory plant communities. According to a seedling survey performed in this study site, thinning provided niches for plant regeneration, wherein species richness and an abundance of native trees increased post-thinning (61). An alteration in carbon input resulted in composition shifts in soil microbial communities and the regulation of belowground carbon flux (15). An increase in and the presence of native trees post-thinning has an impact on fungal communities.

Clear-cut management decreases the biomass of soil fungi and other microbes (2, 49), and has a long-term impact on lodgepole pine soil microbial communities (11, 40). Previous studies reported that thinning has no significant influence on the soil fungal biomass or activity from 4–45 years after thinning (28, 34, 49). Our results demonstrated that thinning significantly influenced the soil fungal community in the short term, but not in the long term. Green tree retention *per se* has a less negative impact than clear cutting when the species richness or abundance of ectomycorrhizal fungi and other taxa is compared (48, 55); green tree retention has been shown to conserve the diversity of mycorrhizal fungi (37, 53). Our results support thinning being a better management method than clear cutting for biodiversity preservation.

After the thinning of *C. japonica* forests, fungal populations increased and recovered within 6 months in plantation soils in Taiwan, whereas soil respiration rates increased and recovered in the fourth year post-thinning in *C. japonica* forests in Japan (51). The study sites in Taiwan and Japan were located at approximately 23°N and 32°N, respectively. Taiwan is located in a tropical area, with higher average temperatures than Japan. Mycelial growth is promoted in warmer and wetter environments (1). Therefore, plant residues in tropical areas have faster decomposition rates than those in temperate areas, and typically return more rapidly to nominal conditions after substrate pulses have been digested. This means that increases in fungal populations in response to thinning may be shorter than the responses observed in temperate forests.

*C. japonica* is an arbuscular mycorrhizal (AM) tree. Few studies have examined the effects of clear cutting and thinning on soil microbial or soil fungal communities in AM tree stands, whereas these effects on ectomycorrhizal trees have been investigated extensively. According to the review by Jones and his co-workers (36), ectomycorrhizal species richness in clear cut treatments was decreased after 1.5, 2, and 4 years; the study sites were located at 51°N, 55°N, and 60°N, respectively (23, 32, 43). These latitudinal differences may influence thinning effects on fungal communities. Furthermore, a longer recovery time was needed in higher latitude areas.

We presume that the effects of clear cutting and thinning on soil microbial communities are slower in colder boreal forests and that fluctuations may be smaller. Thinning had more rapid and short-term effects on the soil microbial community in tropical regions than in temperate and boreal regions. This has implications for forest management in post-thinning conditions, particularly where there is a need for the recruitment or planting of new tree seedlings. Since soil fungal communities have an impact on tree health and productivity, a better understanding of the mechanisms by which these dynamics affect early seedling growth and productivity is needed, which may then lead to the improved sustainability of forestry operations.

## Figures and Tables

**Table 1:** Results of repeated measure design PERMANOVAs testing for effects of season, block, and thinning treatment as well as their interactions on the soil fungal community in a *Cryptomeria japonica* plantation in central Taiwan. A maximum of 999 permutations was possible.

Source	*df*	sum of squares	pseudo *F* ratio	significance (*P* value)
Season	5	162980	11.937	0.0001
Block	3	7829	4.084	0.0001
thinning	2	27366	7.796	0.0007
season × block	15	40975	4.275	0.0001
season × thinning	10	117830	7.835	0.0001
block × thinning	6	10532	2.747	0.0001
season × block × thinning	30	45138	2.355	0.0001
Residuals	143	91368		
Total	214	504670		

**Table 2:** Dissimilarity calculated from binary matrices of DGGE band intensities for soil fungal communities occurring between two seasons in variously thinned plots of a *Cryptomeria japonica* plantation in central Taiwan.

Seasonal comparison	Thinning level		

	0% (control)	25%	50%
spring vs. summer	57.11^a^	58.98	63.32
spring vs. autumn	52.54	49.79	66.45
spring vs. winter	54.53	60.40	57.43
summer vs. autumn	52.43	47.75	50.34
summer vs. winter	57.81	54.52	62.32
autumn vs. winter	57.14	58.17	60.07

a Dissimilarity was calculated according to the method of Kerbs (40).

**Table 3:** Dissimilarity calculated from binary matrices of DGGE band intensities for soil fungal communities occurring in variously thinned plots of a *Cryptomeria japonica* forest in central Taiwan at yearly intervals subsequent to thinning.

	Year		

Thinning comparison	2008	2009	2010
25% vs. 0%	50.33^a^	16.46	12.54
50% vs. 0%	43.81	15.90	15.19
25% vs. 50%	36.55	14.54	18.61

a Dissimilarity was calculated according to the method of Kerbs (40).
